# Histidine Triad Nucleotide-Binding Protein 1 Improves Critical Limb Ischemia by Regulating Mitochondrial Homeostasis

**DOI:** 10.3390/nu15234859

**Published:** 2023-11-21

**Authors:** Tingwen Gao, Shuo Cheng, Hao Lu, Xiao Li, Xinyu Weng, Junbo Ge

**Affiliations:** 1 Department of Cardiology, Zhongshan Hospital, Shanghai Institute of Cardiovascular Diseases, Fudan University, Shanghai 200032, China; 19111210057@fudan.edu.cn (T.G.); lu.hao@zs-hospital.sh.cn (H.L.); li.xiao1@zs-hospital.sh.cn (X.L.); 2 Key Laboratory of Viral Heart Diseases, National Health Commission, Shanghai 200032, China; 3 Key Laboratory of Viral Heart Diseases, Chinese Academy of Medical Sciences, Shanghai 200032, China; 4 National Clinical Research Center for Interventional Medicine, Shanghai 200032, China; 5 Department of Cardiology, Rizhao International Heart Hospital, Rizhao 276825, China; 6 School of Medicine & Holistic Integrative Medicine, Nanjing University of Chinese Medicine, Nanjing 210023, China; chengs@njucm.edu.cn

**Keywords:** critical limb ischemia, neovascularization, histidine triad nucleotide-binding protein 1, endothelial cells, mitochondrial dysfunction, reactive oxygen species

## Abstract

Critical limb ischemia (CLI) is a common complication of diabetes mellitus that typically occurs in the later stages of the disease. Vascularization is indeed an important physiological process involving the formation of new blood vessels from existing ones. It occurs in response to various normal and pathophysiological conditions, and one of its critical roles is to compensate for inadequate oxygen supply, which is often seen in situations like chronic limb ischemia (CLI). Histidine triad nucleotide-binding protein 1 (Hint1) is a member of the Hint family that has been shown to attenuate cardiac hypertrophy, but its role in vascularization still needs to be clarified. In this study, we investigated the role of Hint1 in CLI. We found that Hint1 is significantly reduced in the muscle tissue of STZ-induced diabetic mice and high-glucose (HG)-treated endothelial cells (ECs). Hint1 deletion impaired blood flow recovery and vascularization, whereas Hint1 overexpression promoted these processes. In addition, our in vitro study showed that Hint1 deficiency aggravated mitochondrial dysfunction in ECs, as evidenced by impaired mitochondrial respiration, decreased mitochondrial membrane potential, and increased reactive oxygen species. Our findings suggest that Hint1 deficiency impairs blood perfusion by damaging mitochondrial function and that Hint1 may represent a potential therapeutic target for treating CLI.

## 1. Introduction

Vascularization, the process of forming new blood vessels, is essential for tissue regeneration and repair. It ensures that tissues receive an adequate supply of oxygen and nutrients for their proper functioning and healing [1]. Critical limb ischemia (CLI) is highlighted as one of the most severe forms of peripheral arterial diseases. It is characterized by restricted blood flow to the limbs, leading to serious consequences, including the risk of cardiovascular events like stroke and myocardial infarction, as well as the potential need for amputation [2]. Atherosclerotic vascular occlusions, metabolic syndrome, and diabetes mellitus are identified as well-known risk factors for CLI [3,4,5]. Although the pathological process has been well studied, there are few effective treatment options to improve blood flow in ischemic tissues [6]. Therefore, identifying the crucial regulators of vascularization as therapeutic targets to ameliorate ischemic tissue injury and improve perfusion [7] is of significant importance.

Hint1 (histidine triad nucleotide-binding protein 1) is a highly conserved protein, with a molecular weight of 14 kd, that belongs to the histidine triad (HIT) superfamily containing the His-X-His-X-His-X motif [8]. Recent studies have demonstrated that Hint1 plays crucial roles in neuropsychiatric disorders, including drug addiction, schizophrenia, inherited peripheral neuropathies, and mood disorders [9,10]. Additionally, Hint1 has been identified as a tumor suppressor by regulating multiple molecular mechanisms, such as gene transcription [11], apoptosis [12], and cell cycle control [13]. The deletion of Hint1 has been shown to increase susceptibility to carcinogenesis in mice, further supporting its tumor suppressor role. Furthermore, Hint1 has been implicated in cardiovascular diseases like cardiac hypertrophy. However, whether Hint1 plays a role in vascularization remains unclear.

In the context of limb ischemia, our study delves into the intriguing role of Hint1. We aim to elucidate its significance under both normal physiological conditions and in the presence of hyperglycemia. Employing an animal model of limb ischemia under the background of STZ-induced diabetes mellitus, we thoroughly examine the impact of Hint1 deficiency and endothelium-specific Hint1 overexpression on blood perfusion and capillary formation in mice. This investigation unveils the intricate interplay of Hint1 with the underlying mechanisms, by exploring its effects on blood perfusion, capillary formation, and mitochondrial function. These findings could have significant implications for prognostic assessments and potential therapeutic strategies related to limb ischemia, providing promising avenues for future research and clinical applications.

## 2. Materials and Methods

### 2.1. Experimental Animals

Eight-week-old male mice were procured from Shanghai SLAC Laboratory Animal Co., Ltd. in Shanghai, China, while Hint1 knockout (KO) mice were acquired from CYAGEN in Suzhou, China. Four-week-old male mice were administered intravenous injections of AAV9-Hint1 at a 2 × 10^11^ GC/mouse dose, supplied by OBIO in Shanghai, China. This injection aimed to induce the overexpression of the target protein in the endothelial cells. After a four-week interval post-injection, the mice underwent experimental procedures. To induce diabetic hyperglycemia, intraperitoneal injections of 50 mg/kg streptozotocin (STZ) from Sigma-Aldrich were administered for five consecutive days in each group [14]. Twelve weeks after the STZ induction to diabetes mellitus, the mice underwent femoral artery ligation, following the method described in [15]. The mice were classified into two distinct groups: Control (n = 6) and STZ (n = 6). The mice were anesthetized using 1% pentobarbital sodium for the surgical procedure. The left femoral artery was ligated at three points: the entry point through the inguinal ligament, the middle of the popliteal artery, and the saphenous artery. Small branches were cauterized, and the artery segment between the ligatures was excised. The mice were classified into four distinct groups in the genetical inhibition of Hint1 experiment: WT (n = 5), Hint1 KO (n = 5), WT + STZ (n = 5), and Hint1 KO + STZ (n = 5). The mice were classified into four distinct groups in the overexpression of Hint1 experiment: WT (n = 5), AAV−Hint1 (n = 5), WT + STZ (n = 5), and AAV−Hint1 + STZ (n = 5).

Additionally, the tissue connection between the skin and muscle was disrupted. Blood flow measurements were taken immediately after femoral ligation and at 3 days, 1 week, 2 weeks, and 3 weeks using a Laser Doppler System from Perimed in Sweden (Red indicates bener recovery of hind limb perfusion, while blue indicates poor recovery of hind limb perfusion). The ischemic to non-ischemic hindlimb blood flow ratio was calculated for each animal at each time point. The mice were housed in the Animal Core Facility of Zhongshan Hospital, Fudan University, and were maintained in a 12 h light/dark cycle with access to standard food. All animal procedures were overseen by the Animal Care and Use Committee of Zhongshan Hospital, Fudan University.

### 2.2. Flow Cytometry

A previously established method [16] was employed to generate single-cell suspensions from ischemic muscle tissue. The procedure began by sacrificing the mouse through cervical dislocation, followed by the immediate removal and mincing of the injured gastrocnemius muscle in a 2 mL tube placed on ice. The minced muscle fragments were then subjected to incubation in a PBS buffer containing dispase II (D4693, Burlington, MA, USA) and collagenase IV (17104019, Thermo Fisher Scientific, Lenexa, KS, USA) at 37 °C while being agitated on a shaker (MQD-S2R, SHANGHAI MINQUAN Instrument Co., Ltd., Shanghai, China) for 40 min. Upon completion of the digestion process, the reaction was halted by adding an equal volume of HBSS supplemented with 20% FBS. The resulting suspension was filtered through a 70 μm cell strainer (352350, Corning Falcon, NY, USA) and then through a 40 μm cell strainer (352340, Corning Falcon, NY, USA). The cell suspensions were further processed via centrifugation (400· *g*, 4 °C, 5 min), followed by resuspension in 1 × staining buffer (554656, BD Pharmingen, San Diego, CA, USA), and transferred to a 1.5 mL EP tube. These obtained single-cell suspensions were then utilized for flow cytometry analysis.

Before the surface marker staining step, Fcγ receptors were blocked using a CD16/CD32 monoclonal antibody (FRC-4G8) (diluted at 1:50, MFCR00-4, Thermo Fisher Scientific, Lenexa, KS, USA) for 15 min at 4 °C. Subsequently, the single-cell suspensions were incubated with live/dead-BV780 (diluted at 1:1000, 565388, BD Pharmingen, San Diego, CA, USA) at room temperature for 10 min. Following this, the cells were incubated with fluorophore-conjugated antibodies at 4 °C for 30 min. The antibodies used for flow cytometry included CD45-BUV395 (564616, BD Pharmingen, San Diego, CA, USA), CD11b-BUV737 (564443, BD Pharmingen, San Diego, CA, USA), CD64-BV421 (139309, BioLegend, San Diego, CA, USA), Ly6G-BV510 (127633, BioLegend, San Diego, CA, USA), CD206-APC (17-2061-82, eBioscience, San Diego, CA, USA), and CD31-PerCP-Cy5.5 (562861, BD Pharmingen, San Diego, CA, USA). Flow cytometric analysis was conducted using a BD LSRFortessa X-20 flow cytometer (BD Pharmingen, San Diego, CA, USA), and the resulting data were analyzed with FlowJo 10 software (Tree Star).

### 2.3. Cell Culture

Human umbilical vein endothelial cells (HUVECs) were isolated following a previously described method [17]. They were cultured in complete EC medium (ScienCell, Carlsbad, CA, USA) supplemented with endothelial growth factor, 10% (*v*/*v*) fetal bovine serum (FBS; Gibco, Carlsbad, CA, USA), and 1% penicillin/streptomycin.

### 2.4. Mitotracker ROS Staining

To prepare staining solutions of Mitotracker ROS (C1048, Beyotime, Shanghai, China), we used the following method: Dilute the 1 mM Mitotracker ROS stock solution to the desired working concentration in the appropriate buffer or growth medium. Once HUVECs have reached the desired confluency, remove the media from the dish and add prewarmed (37 °C) staining solution containing the Mitotracker probe. Incubate the cells with the staining solution for 15 to 45 min. Afterward, replace the staining solution with fresh prewarmed buffer and centrifuge the cells to obtain a cell pellet. Carefully aspirate the supernatant and gently resuspend the cells in a prewarmed (37 °C) staining solution containing the Mitotracker probe for 15 to 45 min. Once the cells have been completely stained, wash them in fresh prewarmed buffer. To fix the cells, incubate them with 3.7% formaldehyde in a complete growth medium at 37 °C for 15 min. After fixation, rinse the cells multiple times in the buffer.

### 2.5. MitoSOX Fluorescence Staining

To prepare the MitoSOXTM reagent, we used the following method: Dissolve the MitoSOXTM mitochondrial superoxide indicator (M36008, Invitrogen, Carlsbad, CA, USA) in dimethylsulfoxide (DMSO) to create the MitoSOXTM reagent stock solution. Then, dilute the MitoSOXTM reagent stock solution in a suitable buffer to obtain the MitoSOXTM reagent work solution. Next, apply 1.0–2.0 mL of the 5 μM MitoSOXTM reagent working solution to cover the cells adhering to coverslips. Incubate the cells for 10 min at 37 °C while protecting them from light. Following incubation, gently wash the cells three times with a warm buffer. If desired, stain the cells with counterstains and mount them in the warm buffer for imaging purposes.

### 2.6. Measurements of Oxygen Consumption Rate

The Seahorse Bioscience metabolic analyzer was utilized for assessing the oxygen consumption rate (OCR) of HUVECs, following the manufacturer’s guidelines [18]. HUVECs were initially plated at a density of 2000 cells per well in a Seahorse Bioscience XFe24 cell culture microplate (50-995-965, Agilent Technologies, Carlsbad, CA, USA). Following the baseline measurement, a series of compounds were added sequentially in the following order: oligomycin (Sigma-Aldrich, St. Louis, MO, USA) at a concentration of 1.5 μM, acting as an inhibitor of ATP synthase; cyanide 4-(trifluoromethoxy) phenylhydrazone (FCCP) (Sigma-Aldrich, St. Louis, MO, USA) at a concentration of 0.5 μM, functioning as an uncoupling agent; and a combination of antimycin/rotenone (Sigma-Aldrich, St. Louis, MO, USA) at a concentration of 1 μM. The Seahorse XFp Cell Mito Stress Test was employed to determine critical parameters of mitochondrial function, encompassing basal mitochondrial respiration, ATP-linked respiration, maximal respiration, and spare respiratory capacity [19].

### 2.7. Western Blotting

The protein extraction and Western blotting procedures followed a previously described protocol [20]. Briefly, the cells were washed with PBS and then lysed in ice-cold RIPA buffer (P0013B, Beyotime, Shanghai, China) supplemented with Protease Inhibitor Cocktail (11836153001, Roche, Basel, Switzerland). Equal amounts of protein were loaded onto SDS-PAGE and transferred onto a PVDF membrane (1620177, Shanghai, China). The membranes were blocked using 5% (*w*/*v*) BSA in TBST and incubated with the respective primary antibodies: HINT1 (ab124912, Cambridge, MA, USA), SOD2 (PA5-30604, Invitrogen, Carlsbad, CA, USA), CATALASE (#8841, CST, Danvers, MA, USA), CytoC (#4272, CST, Danvers, MA, USA), and β-actin (#3700, CST, Danvers, MA, USA). Afterward, the membranes were incubated with secondary antibodies conjugated with horseradish peroxidase (anti-mouse or anti-rabbit). Signal intensities were quantified using the Image J software (version 1.53t).

### 2.8. Immunofluorescence

Gastrocnemius muscle sections were washed with PBS and then fixed with 4% paraformaldehyde for 20 min at room temperature (RT). Subsequently, the sections were permeabilized using 0.3% Triton X-100 in PBS for 15 min at RT and blocked with 5% (*w*/*v*) BSA in PBS for 30 min at RT. The muscle sections were then incubated overnight at 4 °C with the primary antibody CD31 (AF3628, R&D, Minneapolis, MN, USA). Following primary antibody incubation, the sections were exposed to Alexa Fluor^®^ 594-conjugated secondary antibodies (1:500) (A-11012, LifeTechnologies, Austin, TX, USA) at 37 °C for 30 min. DAPI staining (40727ES10, Yeasen, Shanghai, China) was performed to visualize the cell nuclei. Imaging of the sections was carried out using a confocal microscope (Zeiss LSM 410, Gooingen, Germany).

### 2.9. Tube Formation Assay

To assess capillary tube formation, the Matrigel assay was conducted following previously established methods [21,22]. In each well of a 24-well plate, approximately 200 μL of cold Matrigel^®^ Basement Membrane Matrix (354234, Corning, NY, USA) was added and allowed to solidify at 37 °C for 30 min. Treated HUVECs were then seeded at a density of 1.8 × 104 cells per well and incubated on the Matrigel for 12 h in a humidified 37 °C, 5% CO_2_ incubator. The Image J angiogenesis analyzer was employed to quantify the extent of tube formation. Specifically, the tube length was measured in 2 randomly selected fields from each well (6 fields in total across 3 wells), and the average value was determined for each sample. This process was repeated in five independent experiments.

### 2.10. siRNA-Mediated Gene Knockdown

Endothelial cells (ECs) cultured in 6 cm dishes, 6-well plates, and confocal dishes were transfected with negative control siRNA (sc-37007, Santa Cruz, Dallas, TX, USA) and siRNA-Hint1 (sc-145966, Santa Cruz, Dallas, TX, USA) using Lipofectamine RNAiMAX Reagent (13778150, Thermo Fisher Scientific, Lenexa, KS, USA) following the manufacturer’s instructions.

### 2.11. Statistics

Continuous variables were presented as mean ± standard error of the mean (SEM). For comparing two groups with equal variance, an unpaired two-sided Student’s *t*-test was used; if the data showed unequal variance, a t-test assuming unequal variance was performed. Two-way ANOVA analysis was employed for experiments involving two factors, followed by post hoc analysis using the Tukey method to adjust for multiple comparisons. Statistical significance levels were set at 0.05 (two-sided), unless stated otherwise. GraphPad Prism 8 (La Jolla, CA, USA) was used for all statistical analyses and graph generation.

## 3. Results

### 3.1. Hint1 Expression Is Reduced in STZ-Induced Mouse Muscle and High-Glucose (HG)-Treated Endothelial Cells

**Figure 1:**
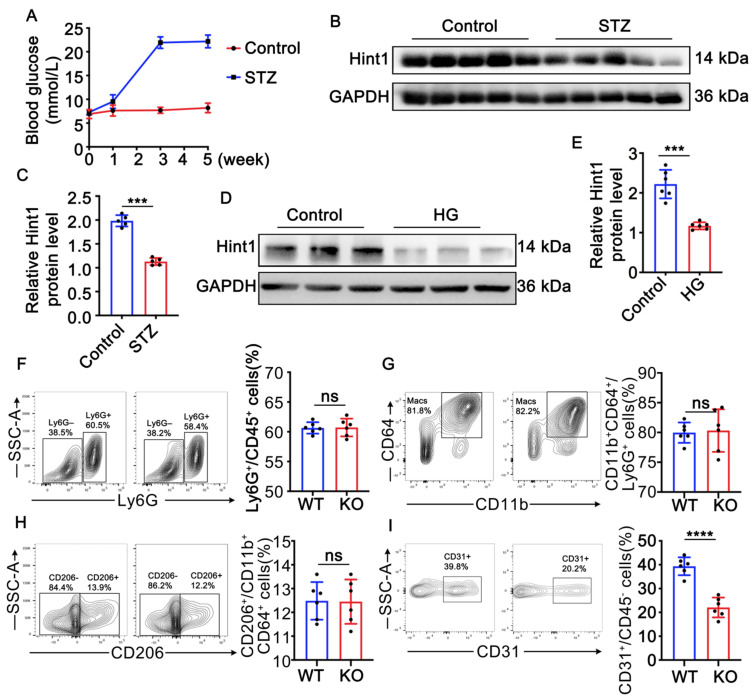
Hint1 is reduced in STZ-induced mouse muscle and high-glucose (HG)-treated endothelial cells. (**A**) Blood glucose levels following STZ injection. (**B**) Immunoblot analysis of Hint1 level in the gastrocnemius muscle from control and STZ-treated mice. (**C**) quantification of Hint1 level. n = 5, unpaired 2-tailed *t* test, *** *p* < 0.001. (**D**) Immunoblot analysis of Hint1 level in the control and HG cultured HUVECs. (**E**) Quantification of Hint1 level. n = 5, unpaired 2-tailed *t* test, *** *p* < 0.001. (**F**) Representative gating plots for Ly6G high cells and percentages of Ly6G high cells within CD45+ cells. n = 6, unpaired 2-tailed *t* test, ns indicates no significant difference. (**G**) Representative gating plots for CD11b+; CD64+ cells and percentages of CD11b+; CD64+ cells within Ly6G+ cells. n = 6, unpaired 2-tailed *t* test, ns indicates no significant difference. (**H**) Representative gating plots for CD206+ macrophages and percentages of CD206+ macrophages within CD11b+; CD64+ cells. n = 6, unpaired 2-tailed *t* test, ns indicates no significant difference. (**I**) Representative gating plots for CD31+ cells and percentages of CD31+ cells within CD45- cells. n = 6, unpaired 2-tailed *t* test, **** *p* < 0.0001.

Diabetic hyperglycemia was induced in mice via an intraperitoneal injection of streptozotocin (STZ) at 50 mg/kg for 5 consecutive days. The STZ-treated mice showed increased blood glucose levels one week after the injection. After 3 weeks of STZ treatment, the mice exhibited a stable diabetic phenotype characterized by sustained high blood glucose levels (Figure 1A). To analyze the effect of diabetes on the expression of Hint1 protein in muscle tissue, gastrocnemius muscle was harvested and subjected to immunoblot analysis. As shown in (Figure 1B,C), the protein level of Hint1 was significantly reduced in the gastrocnemius muscle of the STZ-treated mice, and in vitro experiments with endothelial cells (ECs) also demonstrated that high-glucose (HG) treatment dramatically decreased Hint1 expression (Figure 1D,E). These findings suggest that the downregulation of Hint1 expression is correlated with pathological diabetes and that Hint1 may play a role in the progression of diabetes. Hint1 deficiency barely affects inflammation in ischemic muscle in diabetic mice.

### 3.2. Hint1 Deficiency Barely Affects Inflammation in Ischemic Muscle in Diabetic Mice

As Hint1 has been shown to participate in the regulation of the apoptotic pathway [12], it is likely to be involved in the process of angiogenesis and skeletal muscle regeneration in areas of hindlimb ischemia with ischemic necrosis, where monocytes and macrophages are known to play a critical role [23]. To investigate whether Hint1 deficiency affects the inflammatory condition of ischemic muscle, we performed flow cytometry to measure muscular inflammation. The gating strategy for flow cytometric analysis is shown in Supplementary Figure S1. The results indicated that Hint1 deficiency did not alter the inflammatory state of the ischemic muscle, as evidenced by the percentage of macrophages and M2-polarized macrophages (Figure 1F–H). However, we did observe a significant decrease in CD31-positive cells in Hint1 KO mice (Figure 1I), suggesting that Hint1 may regulate endothelial cells.

### 3.3. Hint1 Deficiency Impairs Blood Flow Recovery

**Figure 2:**
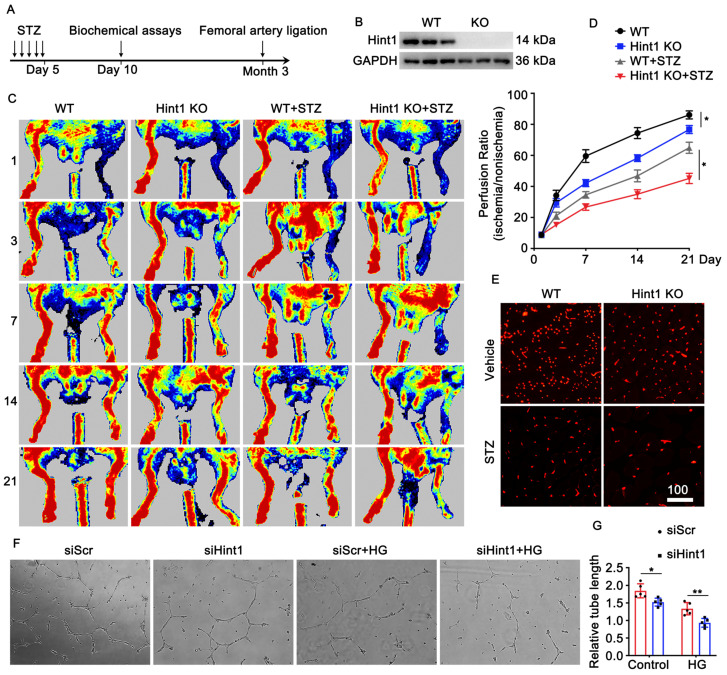
Hint1 deficiency impairs blood flow recovery. (**A**) Experimental protocol. (**B**) Representative immunoblot of Hint1 expression in gastrocnemius muscle from wild-type and Hint1 knockout mice. (**C**) Laser Doppler perfusion images (LDPI) of posterior limb perfusion at 1, 3, 7, 14, and 21 days after femoral artery ligation in each group. n = 5, two-way ANOVA with Bonferroni multiple comparison test. * *p* < 0.05. (**D**) Quantification of perfusion recovery. (**E**) Micrographs of representative hindlimb sections 14 days after surgery; CD31(+) staining is shown in red. Scar bar = 100 μm. (**F**) The ECs were treated with HG (25 mM) or vehicle after transfection with the siHint1 for 24 h, Micrograph of capillary-like tube formation in each group. (**G**) Quantification of tube length. n = 5, two-way ANOVA with Bonferroni multiple comparison test. * *p* < 0.05, ** *p* < 0.01.

To examine the impact of Hint1 on blood flow recovery in chronic ischemic injury related to diabetes, we employed a mouse model of lower limb ischemia, using both Hint1-knockout (KO) and wild-type (WT) mice. The experiment was conducted with and without STZ induction (Figure 2A,B). The perfusion of the ischemic limb was assessed on days 1, 3, 7, 14, and 21 following femoral artery ligation surgery using laser Doppler perfusion imaging. We used Laser Doppler imaging to evaluate blood flow, our results revealed that Hint1-KO mice exhibited a lower perfusion signal than the wild-type (WT) mice in physiological and pathological conditions (Figure 2C,D). In addition, we performed immunofluorescence staining on gastrocnemius muscle sections to assess vascularization (measured using CD31 density). Our findings indicate that CD31 density was lower in Hint1-KO mice than WT mice in both basal and STZ-induced hyperglycemia conditions (Figure 2E). Human umbilical vein endothelial cells (HUVECs) were transfected with siHint1 interference, with or without exposure to a high-glucose medium. Subsequently, a tube formation assay was conducted to assess in vitro angiogenesis. Our findings were consistent with in vivo results, showing that the knockdown of Hint1 inhibited capillary-like tube formation by HUVECs, both under normal and high-glucose conditions. The induction of hyperglycemia by STZ and the treatment with high-glucose levels significantly impacted the recovery of perfusion and the formation of capillary-like tubes (Figure 2F,G). These findings indicate that the absence of Hint1 impairs the restoration of blood perfusion both in physiological and pathological contexts.

### 3.4. Endothelium-Specific Overexpression of Hint1 Improves Perfusion Recovery

**Figure 3:**
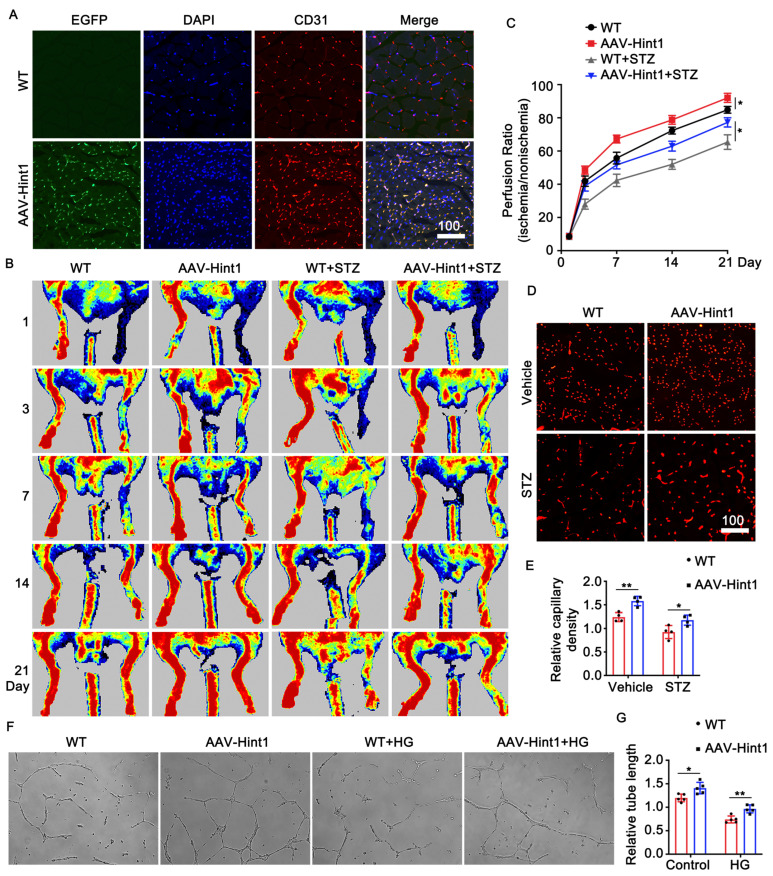
Endothelium-specific overexpression of Hint1 improves perfusion recovery. (**A**) Specific expression of EGFP-tagged Hint1 in endothelial cells in gastrocnemius muscles. Scar bar = 100 μm. (**B**) Laser Doppler perfusion images (LDPI) of posterior limb perfusion at 1, 3, 7, 14, and 21 days after femoral artery ligation in each group. (**C**) Quantification of perfusion recovery. n = 5, two-way ANOVA with Bonferroni multiple comparison test. * *p* < 0.05. (**D**) Micrographs of representative hindlimb sections 14 days after surgery; CD31(+) staining is shown in red. Scar bar = 100 μm. (**E**) Capillary density analysis. n = 5, two-way ANOVA with Bonferroni multiple comparison test. * *p* < 0.05, ** *p* < 0.01. (**F**) Micrograph of capillary-like tube formation in each group. (**G**) Quantification of tube length. n = 5, two-way ANOVA with Bonferroni multiple comparison test. * *p* < 0.05, ** *p* < 0.01.

To investigate the impact of Hint1 on limb ischemia, we administered AAV9 vectors carrying the Hint1 gene under the Tie2 promoter (AAV9-Tie2-Hint1-EGFP) through tail vein injection in mice. Subsequently, we induced STZ and femoral artery ligation. Immunofluorescence staining was performed to confirm the overexpression efficiency of Hint1 in endothelial cells (ECs) (Figure 3A). The overexpression of Hint1, specifically in endothelial cells, led to a significant improvement in the recovery of limb ischemia, as demonstrated by an increase in perfusion signal detected by Laser Doppler imaging evaluation (Figure 3B,C) and capillary density (Figure 3D,E) under both basal and STZ-induced hyperglycemic conditions. To assess the effect of Hint1 on vascularization in vitro, endothelial cells (ECs) were transduced with adenovirus to overexpress Hint1. The results showed that Hint1 overexpression in ECs promoted the formation of capillary-like tubes in both standard and high-glucose media (Figure 3F,G). These findings suggest that the overexpression of Hint1, specifically in the endothelium, may mitigate limb ischemia in vivo and in vitro.

### 3.5. The Silencing of Hint1 Damages Mitochondrial Function in ECs

**Figure 4:**
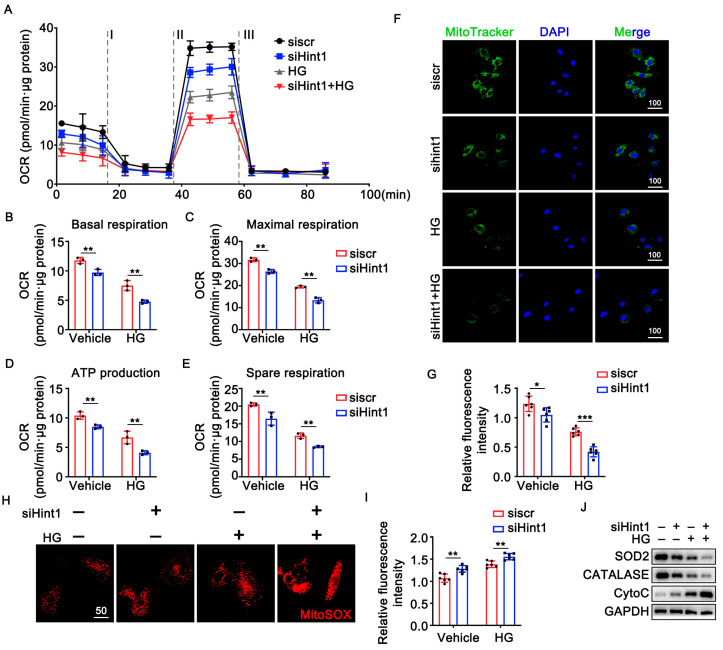
Silencing of Hint1 damages mitochondrial function in EC. (**A**) An analysis of O_2_ consumption in HUVECs using different inhibitors. The ECs were treated with HG (25 mM) or vehicle after transfection with the siHint1 for 24 h. Graphs present the basal OCR (**B**), maximal OCR (**C**), ATP production (**D**), and spare respiratory capacity OCR (**E**) in ECs; n = 3 per group, two-way ANOVA with Bonferroni multiple comparison test. ** *p* < 0.01. (**F**,**G**) MitoTracker staining was used to detect mitochondrial membrane potential (Δψm); Scar bar = 100 μm, n = 5 per group, two-way ANOVA with Bonferroni multiple comparison test. * *p* < 0.05, *** *p* < 0.001. (**H**,**I**) mitoSOX fluorescence staining was used to detect mitochondrial reactive oxygen species (mROS); Scar bar = 50 μm, n = 5 per group, two-way ANOVA with Bonferroni multiple comparison test. ** *p* < 0.01. (**J**) Representative Western blot analysis of SOD2, CATALASE, and CytoC in ECs are shown.

Hint1 is involved in mitochondrial-related oxidative stress and apoptosis [23]. Hint2, an isomer of Hint1, is an important mitochondrial enzyme with similar substrate specificities with Hint1 [24]. Based on our findings, we propose that Hint1 may regulate mitochondrial function, which may improve vascularization. To test this hypothesis, we transfected human umbilical vein endothelial cells (HUVECs) with siHint1 for 24 h and cultured them in either standard or high-glucose (HG) medium. We used a seahorse assay to assess mitochondrial function to measure basal and maximal oxygen consumption rate, ATP production, and spare respiratory capacity in each group. Our results showed that Hint1 knockdown significantly decreased all of these parameters in the standard and HG media. These findings suggest that Hint1 is an important regulator of mitochondrial function in HUVECs and could improve vascularization (Figure 4A–E). Furthermore, the knockdown of Hint1 resulted in a decrease in mitochondrial membrane potential (Δψm), as shown in Figure 4F,G. There was also an increase in mitochondrial reactive oxygen species (mROS) observed in both standard and HG groups, as demonstrated in (Figure 4H,I). Immunoblot analysis revealed that the downregulation of Hint1 led to a decrease in the levels of antioxidant enzymes SOD2 and catalase and an increase in the level of oxidant protein CytoC in both the control and HG group, as shown in (Figure 4J). These findings indicate that the downregulation of Hint1 can accelerate mitochondria dysfunction in ECs.

### 3.6. Hint1 Overexpression Ameliorates Mitochondrial Dysfunction in ECs

**Figure 5:**
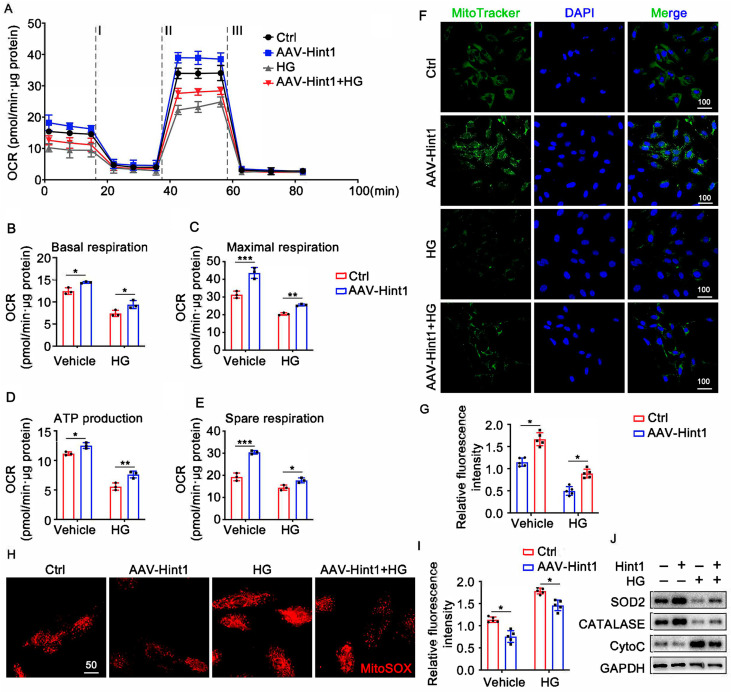
Hint1 overexpression ameliorates mitochondrial dysfunction in ECs. (**A**) An analysis of O_2_ consumption in HUVECs using different inhibitors. The ECs were treated with HG (25 mM) or vehicle after being infected with AAV-Hint1 for 48 h. Graphs present the basal OCR (**B**), maximal OCR (**C**), ATP production (**D**), and spare respiratory capacity OCR (**E**) in ECs; n = 3 per group, two-way ANOVA with Bonferroni multiple comparison test. * *p* < 0.05, ** *p* < 0.01, *** *p* < 0.001. (**F**,**G**) MitoTracker staining was used to detect mitochondrial membrane potential (Δψm); Scar bar = 100 μm, n = 5 per group, two-way ANOVA with Bonferroni multiple comparison test. * *p* < 0.05. (**H**,**I**) mitoSOX fluorescence staining was used to detect mitochondrial reactive oxygen species (mROS); Scar bar = 50 μm, n = 5 per group, two-way ANOVA with Bonferroni multiple comparison test. * *p* < 0.05. (**J**) Representative Western blot analysis of SOD2, CATALASE, and CytoC in ECs are shown.

To gain a deeper insight into the function of Hint1 in facilitating the formation of blood vessels, we conducted experiments where HUVECs were overexpressed with Hint1, both in the presence and absence of HG treatment. Seahorse analysis was performed in HUVECs to study the effect of Hint1 in mitochondrial respiration and found that Hint1 overexpression increased basal and maximal oxygen consumption rate, ATP production, and spare respiratory capacity in both standard and HG media (Figure 5A–E). In addition, we conducted mitoTracker staining and mitoSOX staining to evaluate the mitochondrial membrane potential (ΔΨm) and mitochondrial reactive oxygen species (mROS), respectively. Hint1 overexpression increased mitochondrial membrane potential (Δψm) (Figure 5F,G) and decreased mitochondrial reactive oxygen species (mROS) (Figure 5H,I) in both the standard and HG groups. Furthermore, Western blot analysis showed that Hint1 overexpression increased the levels of antioxidant enzymes, such as SOD2 and catalase, while decreasing the oxidant molecule CytoC levels in both experimental conditions (Figure 5J). These findings prove that Hint1 overexpression can enhance mitochondrial function in endothelial cells, thereby improving their overall health and reducing the risk of dysfunction.

## 4. Discussion

This study demonstrates that Hint1 plays a crucial protective role against limb ischemia in normal and hyperglycemic conditions. Our results show that Hint1 expression is significantly reduced in STZ-induced mouse gastrocnemius muscle and HG-treated ECs. However, we found that endothelium-specific overexpression of Hint1 can enhance perfusion recovery after limb ischemia. Further investigation revealed that Hint1 maintains mitochondrial homeostasis and energy metabolism in endothelial cells, contributing to its protective effect against limb ischemia. Our findings expand our knowledge of the Hint1 function and highlight its novel role in protecting against limb ischemia by maintaining mitochondrial homeostasis.

HINT1 has recently been identified as a tumor suppressor that effectively hinders the development of various cancers in mice, including ovarian, gastric, breast, and liver cancers. Research has shown that reduced levels of HINT1 in liver and gastric cancer may be linked to the hyper-methylation of the Hint1 promoter region [25]. When HINT1 is overexpressed in SW480 and McF-7 tumor cells, it leads to a decrease in Bcl-2 levels, an increase in Bax levels, and elevated expression of caspase-9, caspase-8, and caspase-3, ultimately resulting in the induction of apoptosis in tumor cells [12].

Furthermore, Hint1 is known to regulate the ubiquitination of cyclin-dependent kinase inhibitor 1B (p27KIP1), disrupting the cell cycle [26] and inhibiting the expression of cyclin D1, which in turn suppresses cell proliferation [27]. The interaction between Hint1 and cyclin-dependent kinase Cdk7 lengthens the cell cycle and reduces cell proliferation [28]. These findings collectively suggest that Hint1 plays a crucial role in restraining cell proliferation and promoting apoptosis.

However, the precise role of Hint1 in limb ischemia has remained unclear. Our data demonstrate that Hint1 deficiency negatively affects blood perfusion and capillary formation, both in vivo and in vitro. Moreover, the specific overexpression of Hint1 in endothelial cells using AAV9 was shown to enhance blood flow restoration and angiogenesis, indicating that Hint1 serves as a protective factor against limb ischemia.

Angiogenesis, defined as the process of generating new blood vessels [29,30], encompasses the growth of existing endothelial cells to create fresh capillaries and the regeneration of blood vessels through endothelial progenitor cells [31]. The accurate execution of endothelial angiogenesis relies significantly on proper mitochondrial function and biogenesis [32]. While mitochondrial energy production may be impaired in endothelial cells, other vital mitochondrial functions play pivotal roles in maintaining vascular equilibrium and regulating angiogenesis. These functions encompass the generation of mitochondrial reactive oxygen species (ROS), metabolic control, intracellular calcium regulation, and apoptosis [32]. Endothelial mitochondria possess substantial bioenergy reserves and are essential for mitigating oxidative stress [33]. Even under reduced glycolytic rates, endothelial cells retain the capability to switch to oxidative metabolism by utilizing fatty acids, glucose, and amino acids [34]. Impaired neovascularization in aged POLGA mutant mice underscores the necessity for fully functional mitochondria during angiogenesis. This model illustrates that defects in mitochondrial DNA polymerase result in mitochondrial dysfunction and aging, causing a severe impairment in growth factor-induced angiogenesis without affecting established vascular networks [35]. In POLGA mice, angiogenesis induced by growth factors experiences significant impairment, with no adverse impact on existing vascular networks, reinforcing the importance of mitochondrial function in neovascularization. Growth factors, potent stimulants of angiogenesis, also promote the production of nitric oxide (NO) [36]. NO influences various signaling pathways related to angiogenesis, with its primary target being mitochondrial cytochrome C oxidase, the terminal enzyme of the mitochondrial respiratory chain [37].

Hint1 participated in mitochondrial-related oxidative stress and apoptosis [23]. Hint2 is an isomer of Hint1 that shares similar substrate specificities with Hint1 [24]. Hint2 is an essential mitochondrial enzyme with a characteristic HISI-X-HISI-X-HISI-X-X active site motif, where X is a hydrophobic residue and the second histidine plays a role catalytic nucleophilic role [38,39]. In combination with previous studies, our data further support the notion that Hint1 has a role in mitochondrial function in endothelial cells.

This study has several limitations. Firstly, we did not include samples from patients with lower limb peripheral vascular disease, which limited the clinical perspectives of our findings. Secondly, our focus was solely on the impact of the Hint1 gene on lower limb ischemic phenotypes, necessitating further in-depth mechanistic studies. Lastly, the use of a left femoral artery ligation model in diabetic mouse to simulate lower limb ischemia may not fully replicate the true disease situation, indicating the need for ongoing improvements to enhance our research.

## 5. Conclusions

Our study reveals a substantial reduction in Hint1 expression in the gastrocnemius muscle of streptozotocin (STZ)-induced mice. Importantly, we provide novel evidence demonstrating the critical role of Hint1 in enhancing ischemic blood perfusion by preserving mitochondrial function in endothelial cells under both normal and hyperglycemic conditions. The significant impact of Hint1 on maintaining endothelial cell mitochondrial function highlights its potential as a promising therapeutic target for the treatment of limb ischemia. By targeting Hint1, interventions aimed at preserving or restoring mitochondrial function may hold great promise for improving outcomes in patients with limb ischemia. Further investigations are warranted to fully understand the underlying mechanisms and to explore the translational potential of Hint1 as a therapeutic target in the clinical management of limb ischemia.

## Data Availability

All data are provided within the main text and Supplemental Document.

## Supplementary Materials

The following supporting information can be downloaded at: https://www.mdpi.com/article/10.3390/nu15234859/s1, Figure S1: Gating strategy for the identification of macrophages, M2 polarized macrophages and endothelial cells in the ischemic muscle.

## References

[1] Bussolino F., Mantovani A., Persico G. (1997). Molecular mechanisms of blood vessel formation. Trends Biochem. Sci..

[2] Murabito J.M., Evans J.C., Nieto K., Larson M.G., Levy D., Wilson P.W. (2002). Prevalence and clinical correlates of peripheral arterial disease in the Framingham Offspring Study. Am. Heart J..

[3] Schanzer A., Conte M.S. (2010). Critical limb ischemia. Curr. Treat. Options Cardiovasc. Med..

[4] Faglia E., Clerici G., Clerissi J., Gabrielli L., Losa S., Mantero M., Caminiti M., Curci V., Lupattelli T., Morabito A. (2006). Early and five-year amputation and survival rate of diabetic patients with critical limb ischemia: Data of a cohort study of 564 patients. Eur. J. Vasc. Endovasc. Surg..

[5] Chi C.-Y., Wang J., Lee S.-Y., Chao C.-T., Hung K.-Y., Chien K.-L. (2023). The Impact of Glucose-Lowering Strategy on the Risk of Increasing Frailty Severity among 49,519 Patients with Diabetes Mellitus: A Longitudinal Cohort Study. Aging Dis..

[6] Gupta R., Tongers J., Losordo D.W. (2009). Human studies of angiogenic gene therapy. Circ. Res..

[7] Norgren L., Hiatt W.R., Dormandy J.A., Nehler M.R., Harris K.A., Fowkes F.G.R. (2007). Inter-Society Consensus for the Management of Peripheral Arterial Disease (TASC II). J. Vasc. Surg..

[8] Brenner C., Garrison P., Gilmour J., Peisach D., Ringe D., Petsko G.A., Lowenstein J.M. (1997). Crystal structures of HINT demonstrate that histidine triad proteins are GalT-related nucleotide-binding proteins. Nat. Struct. Biol..

[9] Zimoń M., Baets J., Almeida-Souza L., De Vriendt E., Nikodinovic J., Parman Y., Battaloǧlu E., Matur Z., Guergueltcheva V., Tournev I. (2012). Loss-of-function mutations in HINT1 cause axonal neuropathy with neuromyotonia. Nat. Genet..

[10] Liu P., Liu Z., Wang J., Ma X., Dang Y. (2017). HINT1 in Neuropsychiatric Diseases: A Potential Neuroplastic Mediator. Neural Plast..

[11] Razin E., Zhang Z.C., Nechushtan H., Frenkel S., Lee Y.-N., Arudchandran R., Rivera J. (1999). Suppression of microphthalmia transcriptional activity by its association with protein kinase C-interacting protein 1 in mast cells. J. Biol. Chem..

[12] Weiske J., Huber O. (2006). The histidine triad protein Hint1 triggers apoptosis independent of its enzymatic activity. J. Biol. Chem..

[13] Cen B., Li H., Weinstein I.B. (2009). Histidine triad nucleotide-binding protein 1 up-regulates cellular levels of p27KIP1 by targeting ScfSKP2 ubiquitin ligase and Src. J. Biol. Chem..

[14] Biscetti F., Straface G., De Cristofaro R., Lancellotti S., Rizzo P., Arena V., Stigliano E., Pecorini G., Egashira K., De Angelis G. (2010). High-mobility group box-1 protein promotes angiogenesis after peripheral ischemia in diabetic mice through a VEGF-dependent mechanism. Diabetes.

[15] Caporali A., Meloni M., Miller A.M., Vierlinger K., Cardinali A., Spinetti G., Nailor A., Faglia E., Losa S., Gotti A. (2012). Soluble ST2 is regulated by p75 neurotrophin receptor and predicts mortality in diabetic patients with critical limb ischemia. Arter. Thromb. Vasc. Biol..

[16] Liu J., Pan L., Hong W., Chen S., Bai P., Luo W., Sun X., He F., Jia X., Cai J. (2022). GPR174 knockdown enhances blood flow recovery in hindlimb ischemia mice model by upregulating AREG expression. Nat. Commun..

[17] Ferro A., Queen L.R., Priest R.M., Xu B., Ritter J.M., Poston L., Ward J.P.T. (1999). Activation of nitric oxide synthase by β2-adrenoceptors in human umbilical vein endothelium in vitro. Br. J. Pharmacol..

[18] van der Windt G.J., Chang C., Pearce E.L. (2016). Measuring Bioenergetics in T Cells Using a Seahorse Extracellular Flux Analyzer. Curr. Protoc. Immunol..

[19] Miguel V., Ramos R., García-Bermejo L., Rodríguez-Puyol D., Lamas S. (2020). The program of renal fibrogenesis is controlled by microRNAs regulating oxidative metabolism. Redox Biol..

[20] Liu Z., Han Y., Li L., Lu H., Meng G., Li X., Shirhan M., Peh M.T., Xie L., Zhou S. (2013). The hydrogen sulfide donor, GYY4137, exhibits anti-atherosclerotic activity in high fat fed apolipoprotein E−/−mice. Br. J. Pharmacol..

[21] Arnaoutova I., Kleinman H.K. (2010). In vitro angiogenesis: Endothelial cell tube formation on gelled basement membrane extract. Nat. Protoc..

[22] Yin K.-J., Olsen K., Hamblin M., Zhang J., Schwendeman S.P., Chen Y.E. (2012). Vascular endothelial cell-specific MicroRNA-15a inhibits angiogenesis in hindlimb ischemia. J. Biol. Chem..

[23] Liu F., Dong Y.-Y., Lei G., Zhou Y., Liu P., Dang Y.-H. (2021). HINT1 Is Involved in the Chronic Mild Stress Elicited Oxidative Stress and Apoptosis through the PKC ε/ALDH-2/4HNE Pathway in Prefrontal Cortex of Rats. Front. Behav. Neurosci..

[24] Strom A., Tong C.L., Wagner C.R. (2020). Histidine triad nucleotide-binding proteins HINT1 and HINT2 share similar substrate specificities and little affinity for the signaling dinucleotide Ap4A. FEBS Lett..

[25] Zhang Y.-J., Li H., Wu H.-C., Shen J., Wang L., Yu M.-W., Lee P.-H., Weinstein I.B., Santella R.M. (2009). Silencing of Hint1, a novel tumor suppressor gene, by promoter hypermethylation in hepatocellular carcinoma. Cancer Lett..

[26] Cen B., Deguchi A., Weinstein I.B. (2008). Activation of protein kinase g Increases the expression of p21CIP1, p27KIP1, and histidine triad protein 1 through Sp1. Cancer Res..

[27] Weiske J., Huber O. (2005). The histidine triad protein Hint1 interacts with Pontin and Reptin and inhibits TCF–β-catenin-mediated transcription. J. Cell Sci..

[28] Bieganowski P., Garrison P.N., Hodawadekar S.C., Faye G., Barnes L.D., Brenner C. (2002). Adenosine monophosphoramidase activity of Hint and Hnt1 supports function of Kin28, Ccl1, and Tfb3. J. Biol. Chem..

[29] Folkman J. (2006). Angiogenesis. Annu. Rev. Med..

[30] Ren S.-C., Chen X., Gong H., Wang H., Wu C., Li P.-H., Chen X.-F., Qu J.-H., Tang X. (2022). SIRT6 in Vascular Diseases, from Bench to Bedside. Aging Dis..

[31] Risau W., Flamme I. (1995). Vasculogenesis. Annu. Rev. Cell Dev. Biol..

[32] Marcu R., Zheng Y., Hawkins B.J. (2017). Mitochondria and Angiogenesis. Adv. Exp. Med. Biol..

[33] Dranka B.P., Hill B.G., Darley-Usmar V.M. (2010). Mitochondrial reserve capacity in endothelial cells: The impact of nitric oxide and reactive oxygen species. Free Radic. Biol. Med..

[34] Krützfeldt A., Spahr R., Mertens S., Siegmund B., Piper H. (1990). Metabolism of exogenous substrates by coronary endothelial cells in culture. J. Mol. Cell. Cardiol..

[35] Coutelle O., Hornig-Do H., Witt A., Andree M., Schiffmann L.M., Piekarek M., Brinkmann K., Seeger J.M., Liwschitz M., Miwa S. (2014). Embelin inhibits endothelial mitochondrial respiration and impairs neoangiogenesis during tumor growth and wound healing. EMBO Mol. Med..

[36] Fukumura D., Gohongi T., Kadambi A., Izumi Y., Ang J., Yun C.-O., Buerk D.G., Huang P.L., Jain R.K. (2001). Predominant role of endothelial nitric oxide synthase in vascular endothelial growth factor-induced angiogenesis and vascular permeability. Proc. Natl. Acad. Sci. USA.

[37] Cleeter M.W., Cooper J.M., Darley-Usmar V.M., Moncada S., Schapira A.H. (1994). Reversible inhibition of cytochrome c oxidase, the terminal enzyme of the mitochondrial respiratory chain, by nitric oxide: Implications for neurodegenerative diseases. FEBS Lett..

[38] Brenner C., Bieganowski PPace H.C., Huebner K. (1999). The histidine triad superfamily of nucleotide-binding proteins. J. Cell Physiol..

[39] Mootha V.K., Bunkenborg J., Olsen J.V., Hjerrild M., Wisniewski J.R., Stahl E., Bolouri M.S., Ray H.N., Sihag S., Kamal M. (2003). Integrated analysis of protein composition, tissue diversity, and gene regulation in mouse mitochondria. Cell.

