# Supplementary material for: Histidine Triad Nucleotide-Binding Protein 1 Improves Critical Limb Ischemia by Regulating Mitochondrial Homeostasis
Source: Nutrients. 2023 Nov 21;15(23):4859. doi: 10.3390/nu15234859 (PMC10708213; doi:10.3390/nu15234859)
Supplement: Supplementary file 1 [file nutrients-15-04859-s001.zip › nutrients-2694501-Supplementary.pdf]

Supplementary Material

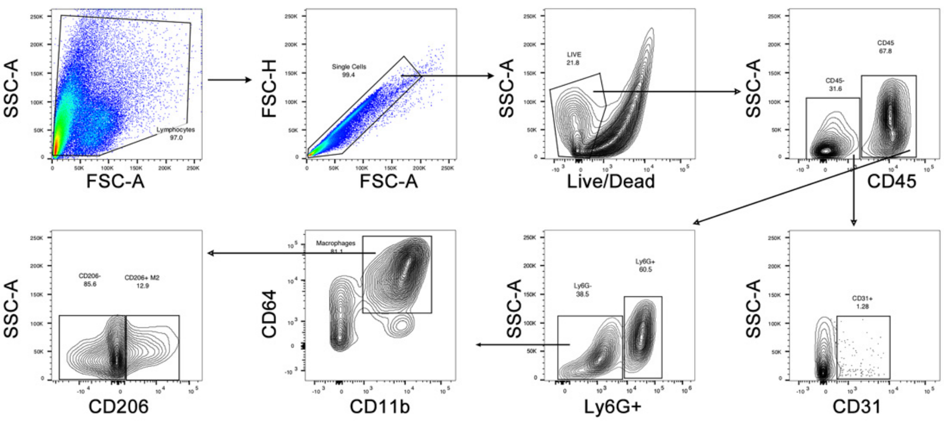

Supplementary Figure S1. Gating strategy for the identification of macrophages, M2 polarized macrophages and endothelial cells in the ischemic muscle. For flow cytometry analysis, single cells isolated from the ischemic muscle were stained with fluorescent conjugated antibodies according to the manufacturer's instructions and then subjected to BD LSRFortessa X-20 analysis.
